# Nurse Manager Practice Environment and Its Influencing Factors: A Multicenter Cross-Sectional Study

**DOI:** 10.1155/2024/6678101

**Published:** 2024-04-23

**Authors:** Yushuang Chen, Xifei He, Qinghua Liu

**Affiliations:** Tongji Hospital, Tongji Medical College, Huazhong University of Science and Technology, Wuhan 430030, China

## Abstract

**Objective:**

This study aimed to evaluate the current status and related factors of practice environments of nurse managers in China.

**Background:**

Insufficient nurse staffing and poor working environment directly increase the burnout and turnover of nurse leaders. Nurse managers play a pivotal role in healthcare organizations, and their performance has been inextricably linked to achieving optimal patient, staff, and healthcare organizational outcomes. However, there are few studies exploring the influencing factors of nurse manager practice environment.

**Methods:**

A cross-sectional study was undertaken to examine a sample of 405 nursing managers who were selected from 10 hospitals located across three provinces in China. The general characteristics questionnaire, the Nurse Manager Practice Environment Scale, the Perceived Stress Scale, and the Career Growth Scale were used. Data were analyzed by descriptive statistics, univariate analysis, and multiple stepwise linear regression.

**Results:**

The total scores of the nurse manager practice environments were 236.71 ± 27.635 (with 270 being the highest possible score), which was at a generally high level. The three lowest scores were adequate budgeted resources, fair and manageable workload, and nurse manager-physician relationships. Nurse manager practice environment was predicted by hospital geographic location, having (or not) training experience in higher ranked hospitals, levels of perceived stress, and career growth scores.

**Conclusion:**

Chinese nursing managers reported a relatively favorable nursing practice environment. Policymakers should pay more attention to the practice environment of nursing managers in small city hospitals, and they could regularly evaluate, monitor, and promote practice environment determinants that are sensitive to disparities between different hospitals. In addition, hospital managers can take action by implementing diversity training programs, developing stress-reduction initiatives, and creating robust career development programs for nurse managers to support nurse managers better. *Implications for nursing management*: a better understanding of the current practice environment of nursing managers is beneficial for improving nursing managers' work environment, which in turn will promote the quality of care delivered and nursing management work. For nurse managers, the characteristics of the management and work environment of the small city hospitals should be benchmarked and learned against the district capital hospitals. Also, hospital administrators were required to adopt strategies to foster psychological support of nurse managers and create pathways and opportunities for professional growth to create a supportive working environment.

## 1. Introduction

In the context global scarcity of nurses, recruiting and retaining nurse managers (NMs) have become a significant challenge [1]. The anticipated vacancy of NMs is alarming, as most NMs are about to retire or resign due to job burnout and dissatisfaction based on baby boomer retirement in the United States [2]. Recent studies have revealed that nurse managers (NMs) have a central role in healthcare organizations, and their performance has been inextricably linked to achieving optimal patient and staff nurse outcomes [3, 4]. Compelling evidence in Saudi, Thailand, and China suggests that improving the quality of the nursing practice environment is considered an important strategy for dealing with the nursing shortage [5–8]. Thus, efforts to improve nurse retention rates must take into account the key role of nurse managers [4].

A good practice environment for NM can stimulate work enthusiasm and promote leadership, which will, in turn, create an environment conducive to supporting nursing staff [9]. Previous studies have been mainly conducted on the practice environments of staff nurses; however, the critical role of nurse managers is ignored [10, 11]. The nurse manager practice environment (NMPE) has been defined as the organizational environment that supports nurse manager practice and influences nurse, patient, and organizational outcomes [12]. Different from the nursing practice environment, NMPE mainly focuses on the role of NMs. Researchers found that the recognition of nurse managers' working environment differs from that of staff nurses [13]. Besides, NMs are indispensable in influencing the professional practice environment of frontline nurses. Thus, it is important to understand the current situation of NMPE. Ensuring a supportive work environment for NMs is the key to retaining these individuals in their roles [14]. Studies also showed that a positive practice environment is critical to job satisfaction, retention, and work performance for NMs [13]. Therefore, nurse managers' work environments warrant the attention of hospital administrators to achieve good workplaces where nurses can focus on patient care.

The established associations between the NMPE and nursing outcomes motivate research to understand the predictors of the NMPE. It is essential to consider a broader range of individual, psychological, and organizational factors, given that the work environment encompasses physical and psychosocial dimensions [12]. Regarding individual factors, there is controversy over the influence of some demographic factors, and more factors remain to be learned. It was reported that sociodemographic differences were considered necessary in evaluating the NMPE [15]. Overall, existing studies indicate the correlation between NMPE with some demographic variables, such as marital status, age, and education [13, 15–17]. Research indicates that hospital characteristics such as location were a significant variable in nurse staff's perception of the practice environment. However, there has been no research exploring the variable among nurse managers.

Few studies have analyzed the associations between nursing managers' perceptions of the practice environment from psychological perspectives. There is growing evidence that mental stress is associated with the practice environment in nurses [18, 19]. Occupational stress is considered a significant factor that negatively influences the practice environment [20]. Nurse managers work in a practice environment that reflects all-day responsibility and often unmanageable workloads, leading to high-stress levels [21]. Previous studies conducted with nursing staff showed that mental strain may affect their self-assessed working environment and the quality of care [18, 22]. Studies revealed that stress has a negative influence on NMPE [17, 23]. However, the abovementioned conclusions stem from a qualitative study and using universal measurement tools. Many findings focus on the staff nurse; the relationship between psychological stress and perceived work environment among NMs still needs further research.

Moreover, recent studies showed that the NMPE is more strongly impacted by organizational characteristics factors such as career development and promotion [4]. Considering the significance of nurses' professional development in assessing the caliber of the nursing practice environment, additional research is required to examine the influence of career development on the working environment of NMs. When organizational characteristics (ongoing development) support NMs' practice, they are more satisfied with their work environment [14, 21, 24]. Career growth is defined as the promotion and development of individual professional ability in the organization and contains career goal, career ability, and career opportunity [25]. It was reported that career development opportunities were the predictors of nurses' work environment [26]. However, there is a lack of evidence that describes career growth in relation to the NMPE.

The importance of nurse managers' practice environments in influencing nursing turnover, direct care, and patient outcomes has been thoroughly studied [17, 27, 28]. The current situation and influencing factors of the NMPE remain to be learned to make international comparisons. Prior studies have been mainly focused on the practice environments of clinical nurses; there is a lack of studies evaluating practice environments from the viewpoint of NMs [11, 28]. Besides, there have been no previous studies that have examined factors influencing NMPE from the perspective of psychological and organizational characteristics using quantitative research. Therefore, the present study aims to investigate the current status of the practice environment and its associated factors among nurse managers in China and to find the aspects that need to be improved the most.

## 2. Methods

### 2.1. Design and Sample

This was a multicenter cross-sectional study performed in August, 2023, in 10 hospitals in Hubei, Shanxi, and Jiangxi provinces in China. A multicenter cross-sectional study was conducted on nurse managers from 10 hospitals across three provinces (Hubei, Shanxi, and Jiangxi) in the central, northern, and southern areas of mainland China. Of the ten hospitals, four are tertiary hospitals located in the provincial capitals Wuhan, Taiyuan, and Nanchang. The remaining six secondary hospitals are located in smaller cities, namely, Suizhou, Shiyan, and Enshi. Cross-sectional designs help determine the current situation of the practice environment for nursing managers and analyze the impact of multiple factors simultaneously, as represented by a study sample. The target population of this study was inpatient and outpatient nurse managers with at least 12 months of experience in the nurse manager role. The study population includes hospitals from provincial capitals such as Taiyuan, Nanchang, and Wuhan, as well as prefecture-level cities such as Suizhou, Shiyan, and Enshi. The convenience sampling method was used in this study. A snowball methodology was used in a convenience sample identified by the director of the Nursing Department, the chief nurse of internal medicine department, and the head nurses. The author confirmed and contacted the nursing department directors and head nurses of 10 hospitals, forwarded the online questionnaire to nursing managers of each hospital, and asked them to voluntarily fill it out. The inclusion criteria were (a) first-line registered nurse manager (head nurse or nursing supervisor) on duty; (b) having worked as nurse manager for more than one year; and (c) having volunteered to participate and provided informed consent. The exclusion criteria were (a) serving in an interim role and (b) nurse managers in administrative positions without managing any patient care areas. In terms of sample size estimation, G Power 3.1 software was used to calculate the sample size needed for this study. The linear multiple regression algorithm was selected. There were 24 variables in this study, including 11 sociodemographic characteristics and 13 scale-associated dimensions. With 95% confidence intervals and 0.8 power, the minimum sample size in our study was 169. Considering a possible 20% wastage rate, a total of 212 participants were needed.

### 2.2. Instrument

#### 2.2.1. General Characteristics Questionnaire

The general characteristics that the questionnaire mainly investigated were the nurse managers' gender, age, marital status, highest education, hospital grade, hospital geographic location, professional title, working years, weekly working hours, teaching work experience, and whether they have training experience in higher ranked hospital.

#### 2.2.2. The Nurse Manager Practice Environment Scale, NMPES

NMPES was designed to describe and assess nurse managers' practice environments by Dr Warshawsky and the Chinese version of NMPES had undergone cross-cultural debugging and validation [29, 30]. NMPES contains 45 items, with a total of 8 dimensions: (1) empowering administrative leaders to create a culture of patient safety (a blame-free environment with established lines of responsibility and accountability), (2) nurse manager-director relationship, (3) culture of generativity (the organization supports ongoing development of nursing leaders), (4) adequate budgeted resources, (5) culture of meaning (the organization's mission and vision are aligned with the organization's mission and vision), (6) NM-physician relationships, (7) NM-unit staff relationships, and (8) fair and controllable workload. Items are measured on a six-point Likert scale ranging from 1 = “completely disagree” to 6 = “completely agree”. Scoring is based on the mean score for the overall scale and mean scores of the subscales. Mean scores close to 6 and 1 suggest that the practice environment is positive or negative (poor), respectively. The higher the score, the better the working environment of the nurse manager. The Chinese version of NMPES has a Cronbach's *α* of 0.917, retest reliability was 0.968, split-half reliability was 0.952, and Content validity was 0.96. The NMPES has a Cronbach's *α* of 0.917 in our study.

#### 2.2.3. Perceived Stress Scale, PSS

PSS is a widely used psychological stress measurement tool. It was developed by DR Cohen and the Chinese version of PSS was translated and validated [31, 32]. It contains 14 items, 2 dimensions, i.e., perceived stress (7 items) and perceived coping ability (7 items), and uses a five-point Likert scale ranging from 0 = “never” to 4 = “always”. The total score on the scale is 0–56 points, and the higher the score, the higher the occupational stress level. The Chinese version of PSS has a Cronbach's *α* of 0.78 and a validity coefficient of 0.73, indicating good reliability and validity.

#### 2.2.4. Career Growth Scale, CGS

CGS was developed by Weng and Xi [33] and contains 15 items with 3 dimensions, namely, career goal, career capacity, and career opportunity. It uses a five-point Likert scale ranging from 1 = “no match at all” to 5 = “very high match”; a higher score indicates a higher level of professional growth status. The CGS has good internal consistency, with Cronbach's *α* of 0.926 for the total scale. Also, CGS was proven to be acceptable, valid, and reliable for the evaluation of nurse career growth in Chinese hospitals [25].

### 2.3. Data Collection and Ethical Considerations

A total of 5 participants were recruited to fill out the online survey questionnaire and provide feedback to ensure that the questionnaire can be understood without difficulty. The online questionnaire link was forwarded to the social media group by the nurse directors or head nurses of each hospital, as requested by the researcher. Participants received the link and were voluntary to fill out the questionnaires on the website platform. If the participants were interested in the study, they could visit the questionnaire and receive a study description at the beginning of the survey via the online link. Participants were required to read and sign an informed consent form before filling out the questionnaire. Only when the participants voluntarily click agree, can they continue to complete the questionnaire survey content. All participants in the survey were anonymous and voluntary, and they were allowed to withdraw from the study at any time. Questionnaires with a filling time of less than 120 seconds were considered invalid questionnaires and were excluded, according to the preliminary experimental results. Two researchers independently exported the results into Microsoft Excel format, checked them, and then imported them into the SPSS software. This study was approved by the Institutional Ethics Review Board of the Tongji Hospital, Tongji Medical Department, Huazhong University of Science and Technology (TJ-IRB20230845).

### 2.4. Statistics Methods

The data were analyzed by using SPSS 26.0. The measurement data all following a normal distribution were described by means and standard deviations. The counting data were described by frequency and percentage. This study used *t*-tests or one-way ANOVA to compare the differences in the practical environment of different groups of nurse managers. Pearson correlation analysis was used to analyze the correlation between nurse manager's practice environments and perceived stress and career growth. Multiple linear regression analysis was used to analyze related factors. The statistical significance was defined as *P* < 0.05.

## 3. Results

### 3.1. Demographics of the Participants

A total of 446 participants were recruited, 405 (90.8%) were valid for analysis. Most of the participants were from the level III hospital (83.7%), located in the district capital (69.6%), female (95.7%), and 31–45 years old (70.6%). Over 60% of the participants had more than 15 years of job experience. Married nursing managers accounted for 93.8% of the participants. As for highest education, 84% of the nursing managers had bachelor's degrees, and only 3.7% of the study samples were in junior college or below. Participants with supervisor nurse and associate professor of nursing or above position titles accounted for 60% and 33.8% of the sample, respectively. In terms of weekly working hours, only 9.9% of participants had 40 hours or less per week; most of the participants had 41 to 59 hours per week. Over 50% of the participants had training experience (Table 1).

### 3.2. Scores of Nurse Manager Practice Environment

The total scores of the investigated nurse manager practice environments were 236.71 ± 27.635, which was at a favorable level, and the specific scores of dimensions are presented in Table 2. NM-unit staff relationships, culture of meaning, and culture of generativity were marked highly, while comparatively lower scores were adequate resources, fair and controllable workload, and NM-physician relationships. In terms of the specific item, the highest-scored item was “maintaining a reputation for excellence is important to the hospital leaders (4.26 ± 1.387)”, and the lowest-scored item was “the budget allocations for my patient care area(s) are adequate (5.77 ± 0.509)”.

### 3.3. Factors Related to Nurse Manager Practice Environment

Independent sample *t*-tests and one-way ANOVA indicated that the total scores of nurse manager practice environment differed significantly among nurses of different hospital grade, hospital location, age, weekly working hours, and further education/training experience (*P* < 0.05) in Table 3.

Furthermore, the results in Table 4 show that the perceived stress score and career growth score had a significant correlation with nurse manager practice environments (*P* < 0.05). Also, the perceived stress and career growth of the nurse managers are at a moderate level. For perceived stress, the highest dimension score was the ability to cope with stressor (mean, 11.39 (SD, 5.28)). The lowest dimension was general stress (mean, 10.70 (SD, 4.29)). For career growth, the highest mean subscale score was career capability. The lowest mean subscale score was career opportunities (Table 4).

### 3.4. Multivariate Analyses of Nurse Manager Practice Environment Scores

A multiple linear regression model was used to avoid confounding factors, and variables that had a significant correlation with the nurse manager practice environment in the one-way ANOVA were taken as independent variables. Also, the stepwise introduction method was introduced into the corresponding regression equation. According to the results of the regression analyses, general stress, career growth, career opportunity, hospital location, and further education/training experience explained 58.6% of the total model variance (Table 5).

## 4. Discussion

This study was a multicenter cross-sectional study that examined the status and influencing factors of the nurse manager practice environment. The results showed that Chinese nursing managers reported a relatively favorable nursing practice environment, but the scores on subscales such as adequate budgeted resources, fair and manageable workload, and NM-physician relationships were still low. The results of the study showed that nurse managers working in hospitals located in small cities, with training experience in higher-ranked hospitals, higher levels of perceived stress, and poor career growth reported higher ratings of their practice environment.

The total score of the nurse manager practice environment was 236.71 (SD, 27.635) (where 270 was the highest possible score). The participants in this study reported a generally high level, which is marginally higher than the total score of NMPES reported in a previous study in China, i.e., 190.35 (SD = 14.66) and 216.13 (SD = 29.42) [30, 34]. These findings are consistent with research showing that NMPES scores were overall reported as moderate [27, 35]. Warshawsky's study reported a moderate to moderately high level among NMs with the same scale in the United States [36]. A survey in Finland showed that NMs experienced more negative perceptions of the practice environment [17]. The possible reason for these differences in results may be different investigation times and areas. We compare the results of the practice environment in prepandemic studies [30, 37], and the results showed some positive changes in the nurse manager practice environment. In the recovery and reconstruction process after the epidemic, a healthy working environment is an important component of our stable nursing team. Within the pandemic environment, managers may feel unsupported with their practice environment [35]. The results of the study indicate that the improvement of the nurse manager practice environments has been achieved. From a policy perspective, the Chinese government introduced a series of relevant policies to ensure the development of the nursing team from various aspects, providing a strong policy foundation for it. For instance, in the National Nursing Development Plan of the China (2021–2025), the importance was stressed to strengthen the construction and development of nursing teams, which optimizes the practical environment for nursing managers.

With regards to the subscales of NMPES, the findings showed that the adequate budgeted resources subscale had the lowest mean (SD) score (4.5 [1.1]), whereas NM-unit staff relationships were the subscale with the highest mean (SD) score (5.5 [0.6]). Overall, these findings are in accordance with findings reported by previous studies [13, 27, 35, 36]. They have demonstrated that the rational allocation and use of resources have become an important and common issue in the context of insufficient personnel and high workload and hospital leaders should align resources to support the creative work of nurse managers. Fair and manageable workload is also a prominent issue in the results of NMPES scores since the score is second only to the adequate budgeted resources. A qualitative study showed that none of the nurse managers reported satisfaction with their workload or work-life balance [38]. Similarly, it is reported that nurse managers experienced significant work pressure, which increases their workload and working hours than other nurse staffs [39]. The results of this study show that a lack of resources and excessive workload were hindering factors that challenged nurse managers' practice environment. The item “the budget allocations for my patient care area(s) are adequate” resulted in the lowest score among all items, reminding us that in the context of poor staffing; managers still face the challenge of improving the level of nursing human resource allocation and achieving dynamic and reasonable allocation of human resources. Healthcare organizations and hospital senior administrators play an important role in promoting a healthy professional practice environment in NMs. Hospital managers should attach importance to the working environment of NMs, make efforts to support, authorize, and lead change to create a practice environment and ultimately further optimize nursing work.

The results of this study indicate that nurse managers from hospitals in the district capital had significantly higher nursing manager practice environment scores than in the small city. The findings are consistent with research showing that hospital geographic location is one of the influencing factors in the professional practice environment of nurse staffs [40]. One similar study conducted on nursing staff indicated that nurses from hospitals in district capitals rated practice environments significantly more highly than nurses from smaller city hospitals [40]. The findings match those observed in earlier studies using focus group interviews that the geographic location can influence how the nurse manager perceives their practice environment [41]. A possible explanation for this might be that nurse managers who worked in hospitals located in the district capital typically had a larger labor pool and more favorable physical environment than other hospitals, making it easier for them to fill vacancies. This finding is supported by another study that found that hospitals located in rural or suburban areas have fewer qualified registered nurses to fill the position of nurse manager and have decreased staffing, which may influence the nurse manager practice environment [42]. Therefore, the geographic location can influence how the nurse manager perceives the practice environment. It was reported that NMs who worked in private hospitals were more likely to evaluate the practice environments positively than those who worked in public or university hospitals [15, 43]. As for hospital grade, this study's results showed no differences in the NMPEs at different levels of hospital. The results also provide evidence that the scores of NMPES of the small city as well as grade II hospital are in a relatively poor state and need to be improved urgently. For nurse managers, the managerial style and work environment of the small city hospitals should be benchmarked against the district capital hospitals. Therefore, it is recommended that hospital managers attach importance to the working environment of nursing managers in small cities by improving the hospital support and guarantee system and providing human resources support and equipment.

In this study, it is interesting to note that participants who had no training experience in higher-ranked hospitals scored highest overall on the nursing manager practice environment, which has not yet been reported before due to a lack of relevant research. The results of this study showed that NMs with training experience in higher ranked hospitals scored statistically significantly lower than those without training experience in terms of resources, workload, and other aspects by further analyzing the subscale score. In contrast, prior study revealed that nurses who have management training experience evaluate their practice environment more positively [15]. Unlike other studies, training experience in higher ranked hospitals referred to in this study does not include academic education but mainly includes training, internships, study visits, or overseas experience for NMs. Hospital rankings are routinely used by medical staffs as a guide in further training in China. The “higher ranked” hospital usually refers to a comprehensive tertiary hospital, an affiliated teaching hospital of a well-known university, or foreign hospital in developed countries. This is probably a consequence of the equipment and system of the superior hospital being more perfect. It should be noted that the perceptions of nursing workplace were associated with having adequate staffing and resources [44]. We speculate that although the training experiences may offer a sense of fulfilment, the expectation of the gap between two different work environments may also decrease satisfaction for nurse managers in their current nursing unit. It was demonstrated in several studies that the different strategies and policies between hospitals will influence the work environment of nurses [45, 46]. This is supported by research that continuing training, usually at a higher-ranked hospital, provides opportunities for nurse managers to update their knowledge and technology, leading to high-level expectations of the practice environment [26]. Also, it seems that when the practice environment of the hospital where they trained is better, the expectations towards the current situation will be affected. However, more research is needed to analyze the differences between expectations and perceptions of the work environment since we did not measure the perceived and expected work environment by NMs. The unexpected findings have extended our knowledge of the perceptions of practice environment in NMs, which are affected by complex aspects. Thus, areas to improve the practice environment of nurse managers include considering the nurse manager's perspective on the current work environment and increasing their involvement in organizational decision-making. More support is provided to those with training experience, and NMs can transform and apply the management experience, organizational culture, and new technologies learned from higher ranked hospitals to help ultimately improve their work environment and nursing work.

The results also revealed that perceived stress had a significant correlation with NMPES. Both dimensions of perceived stress are negatively correlated with the working environment of nursing managers, and general stress is significantly correlated with NMPES. Better nursing manager practice environments are associated with less perceived stress. Previous studies have confirmed the relationship between a favorable environment for nursing practice and a lower occupational stress level [20, 47]. The work environment of nurse managers has been described as stressful and challenging [48]. It was reported that role overload was the primary predictor of work environment stress in nurse managers [17, 49]. A study in the United States also showed that approximately 62% of nursing managers plan to resign within the next 2–5 years due to occupational stress and burnout [50]. Burnout is a mainly negative consequence of stress, which may decrease satisfaction at work, thereby perpetuating unhealthy work environments of NMs [51]. Therefore, the influence of current psychological states on NMPES deserves attention, and more efforts are needed for nurse managers to keep stress in perspective, contributing to developing healthier work environments.

This study indicated that NMPES had a significant positive correlation with career goal, career capability, and career opportunity. Career growth measures the speed of the employee's career/professional development [33]. Career opportunity relates to the feedback and perception of their career development, promotion speed, and salary increase [25]. Several studies have confirmed the influence of career development is strongly positively correlated with nurses' perceptions of their work environment [52]. A quasiexperimental study in a Dutch hospital by Bloemhof indicated that developing multiple career paths for nurses can improve the nurse work environment [53]. Nurse managers need to conduct suitable career plans for every nurse manager and promote career development in organizational environments that support NM practice [36]. The results of Luse's study showed that managers' managerial strategies, such as career ladders can improve the overall working environment [54]. In a cross-sectional study of 1,010 nurses, career opportunities and job satisfaction were significant variables of the self-assessed work environment in Slovenian hospitals [26]. The majority of these variables also explained the NMPES in our study though the research population is different. This study is the first to report the effect of career growth on NMPEs. Creating pathways for personal growth and development for nursing managers and enhancing management skills will improve their work environment.

### 4.1. Limitations

A few limitations were identified in our study. First, the method of convenience sampling and cross-sectional design used in this study may limit the generalization of the results. Although the research subjects involve different levels of hospitals and six regions, the representativeness of the sample will have a particular impact on the results. Second, regarding the data collection, only self-report online surveys were used, and this may result in reporter bias. The use of multiple data collection methods may enrich the findings. Finally, we only explored the influencing factors from the demographic, psychosocial, and organizational aspects. Other organizational and policy factors are also important variables of the nurse manager practice environment. It is recommended that more comprehensive influencing factors need to be explored to explain the nurse manager practice environment based on a mature theoretical framework. Similar studies in the future can conduct stratified random sampling research and carry out longitudinal studies in order to obtain more rigorous and accurate conclusions.

## 5. Conclusions

This study was conducted to investigate the status and influencing factors of the nurse manager practice environment in China. Our results indicated that the Chinese nursing manager practice environment has achieved some improvements with a relatively high score. However, adequate budgeted resources, fair and manageable workload, and NM-physician relationships remain the main issues. Hospital geographic location, training experience in higher ranked hospitals, perceived stress, and career growth were associated factors of the nursing manager's practice environment. It is suggested that more actions need to be taken to improve nursing managers' practice environment by developing a reasonable human resource allocation plan and scientific workflow to increase nursing human resources and material support.

## 6. Implications for Nursing Management

The results of this study provide insight into nurse managers' perceptions of the practice environment and can be used to form recommendations related to strategies to improve the practice environment. Hospital managers should regularly evaluate the status of the nurse manager practice environment and make improvements in areas of weakness to improve the current status.

Moreover, healthcare leaders can promote a front-line nurse manager practice environment by enhancing the predictors of the practice environment. In terms of hospital characteristics (hospital locations), hospital managers need to learn from larger hospitals to improve human resources and the physical environment to equip the workplace in smaller city hospitals. The results of this study suggest that hospitals in small cities may face particular challenges and require more attention in the practice environment. Although geographical location is an unchangeable factor, hospitals can assess, monitor, and promote practice environment determinants that are sensitive to disparities between different hospital geographic locations. Hospital administrators should emphasize the importance of making constructive changes to the practice environment to attract and maintain nurse managers in these hospital settings. In terms of training experience in a higher-ranked hospital, it is possible to take advantage of the experience to improve the status quo of the current unit. Nursing managers' training experience may expand their horizons and can help to provide specific and personalized recommendations to improve the working environment. Training programs and further education opportunities directed at less experienced nurse managers, especially those working at hospitals in small cities, need to be emphasized. Regulations and policies should focus on addressing nurses' expectations and their satisfying factors to positively impact the practice environment and retention. In terms of psychosocial stress and career development, nursing leaders can take measures to reduce work pressure and provide stress management training programs for NMs, which may positively affect the practice environment. Healthcare leaders need to build a platform conducive to the career growth of NMs, provide career management programs, and consider framing these actions as strategies that potentially contribute to improving job satisfaction and nurse retention.

## Figures and Tables

**Table 1:** Sociodemographic data of the investigated nurse managers.

Variables		*N* = 405/means	Percentage/standard deviation
Hospital grade	Grade II hospital	66	16.3
	Grade III hospital	339	83.7

Hospital geographic location	District capital	282	69.6
	Small city	123	30.4

Gender	Male	17	4.2
	Female	388	95.8

Age (years)	≤30	13	3.2
	31–45	286	70.6
	>45	106	26.2

Working years	<5	6	1.5
	6–10	35	8.6
	11–15	103	25.4
	>15	261	64.4

Marital status	Married	380	93.8
	Unmarried	13	3.2
	Widowed/divorced	12	3.0

Highest education	Junior college or below	15	3.7
	Bachelor's degree	340	84.0
	Master's degree and above	50	12.3

Position title	Senior nurse	25	6.2
	Supervisor nurse	243	60
	Associate professor of nursing	130	32.1
	Professor of nursing	7	1.7

Weekly working hours(h)	≤40	40	9.9
	41–59	311	76.8
	≥60	54	13.3

Training experience in higher ranked hospitals	Yes	226	55.8
	No	179	44.2

**Table 2:** Scores of the NMPES.

Variables	Total score (mean ± SD)	Mean score (mean ± SD)	Rank
Nurse manager practice environment (45 items)	236.71 ± 27.635	5.26 ± 0.614	—
Adequate budgeted resources (4 items)	18.19 ± 4.227	4.54 ± 1.056	8
Fair and manageable workload (4 items)	19.84 ± 3.723	4.96 ± 0.931	7
NM-physician relationships (3 items)	15.95 ± 2.179	5.31 ± 0.726	6
Empowering organizational culture of patient safety (15 items)	79.88 ± 9.251	5.32 ± 0.616	5
NN-director relationship (6 items)	32.04 ± 4.475	5.34 ± 0.745	4
Culture of generativity (6 items)	21.92 ± 2.479	5.39 ± 0.659	3
Culture of meaning (4 items)	21.92 ± 2.479	5.47 ± 0.619	2
NM-unit staff relationships (3 items)	16.52 ± 1.803	5.50 ± 0.601	1

**Table 3:** Scores for NM PE with different demographic characteristic.

Variables		Overall scores for nursing manager practice environment (mean ± SD)	*t*/*F* value	*P* value
Hospital grade	Grade II hospital	223.30 ± 34.977	4.404	0.001^*∗*^
	Grade III hospital	239.32 ± 25.212		

Hospital geographic location	District capital	240.17 ± 23.817	3.882	0.001^*∗*^
	Small city	228.77 ± 33.636		

Gender	Male	234.71 ± 23.984	−0.305	0.761
	Female	236.79 ± 27.808		

Age (years)	≤30	226.31 ± 45.256	3.042	0.049^*∗*^
	31–45	238.78 ± 26.704		
	>45	232.40 ± 26.925		

Working years	<5	239.83 ± 30.564	0.054	0.984
	6–10	236.66 ± 37.421		
	11–15	235.99 ± 30.940		
	>15	236.92 ± 24.690		

Marital status	Married	237.50 ± 27.476	2.542	0.080
	Unmarried	224.62 ± 28.915		
	Widowed/divorced	224.75 ± 27.854		

Highest education	Junior college or below	227.67 ± 30.745	0.887	0.413
	Bachelor's degree	236.88 ± 28.112		
	Master's degree and above	238.26 ± 23.053		

Position title	Senior nurse	233.68 ± 35.725	0.361	0.781
	Supervisor nurse	237.59 ± 27.475		
	Associate professor of nursing	235.36 ± 26.199		
	Professor of nursing	241.71 ± 31.245		

Weekly working hours(h)	≤40	239.68 ± 26.185	3.087	0.047^*∗*^
	41–59	237.81 ± 26.576		
	≥60	228.17 ± 33.131		

Training experience in higher ranked hospitals	Yes	232.48 ± 28.819	−3.508	0.001^*∗*^
	No	242.04 ± 25.143		

^*∗*^Statistically significant.

**Table 4:** Correlation between variables and NMPES.

Variables		Range	Mean ± SD	*r*	*P*
Perceived stress		0∼56	22.09 ± 7.235	−0.423	0.001^*∗*^
	General stress	0∼28	10.70 ± 4.290	−0.423	0.001^*∗*^
	The ability to cope with stressors	0∼28	11.39 ± 5.824	−0.213	0.001^*∗*^

Career growth		15∼75	59.45 ± 9.010	0.713	0.001^*∗*^
	Career goal	4∼20	17.06 ± 2.587	0.659	0.001^*∗*^
	Career capability	4∼20	18.06 ± 2.246	0.616	0.001^*∗*^
	Career opportunity	7∼35	24.32 ± 5.836	0.571	0.001^*∗*^

^*∗*^Statistically significant.

**Table 5:** Variables of the multiple linear regression.

Variables	Unstandardized coefficients (b')	Standardized coefficients (Beta)	*t*	*P*
Constant	121.045	—	12.966	0.001^*∗*^
Hospital geographic location	−6.012	−0.100	−2.995	0.003^*∗*^
Training experience in higher ranked hospitals	4.228	0.076	2.274	0.023^*∗*^
General stress	−1.351	−0.210	−6.152	0.001^*∗*^
Career growth	2.944	0.960	12.745	0.001^*∗*^
Career opportunity	−1.776	−0.375	−5.066	0.001^*∗*^

^*∗*^Statistically significant. *R*^2^ = 0.591, adjusted *R*^2^ = 0.586, *F* = 115.309, and *P* < 0.001.

## Data Availability

The cross-sectional study data used to support the findings of this study are available from the corresponding author upon reasonable request.
